# Investigation on the mechanisms of human sperm DNA damage based on the proteomics analysis by SWATH-MS

**DOI:** 10.1186/s12014-022-09391-9

**Published:** 2023-01-06

**Authors:** Chun-Hui Zhu, Ye Wei, Fang Chen, Feng Li, Sheng-Min Zhang, Nai-Jun Dong, Tong-Min Xue, Kai-Feng Liu, Heng-Mi Cui, Jin-Chun Lu

**Affiliations:** 1 Center for Reproductive Medicine, Northern Jiangsu People’s Hospital Affiliated to Yangzhou University/Clinical Medical College, Yangzhou University, Yangzhou, 225001 Jiangsu China; 2 Institute of Epigenetics and Epigenomics, College of Animal Science and Technology, Yangzhou University, 48 Wenhui Road, Yangzhou, 225009 Jiangsu China; 3 Center for Reproductive Medicine, Zhongda Hospital, Southeast University, 3 Xinmofan Road, Nanjing, 210037 Jiangsu China

**Keywords:** Proteomics, Sperm DNA damage, Male infertility, Biomarker

## Abstract

**Background:**

Spermatozoa have the task of delivering an intact paternal genome to the oocyte and supporting successful embryo development. The detection of sperm DNA fragmentation (SDF) has been emerging as a complementary test to conventional semen analysis for male infertility evaluation, but the mechanism leading to SDF and its impact on assisted reproduction remain unclear. Therefore, the study identified and analyzed the differentially expressed proteins of sperm with high and low SDF.

**Methods:**

Semen samples from men attended the infertility clinic during June 2020 and August 2020 were analyzed, and sperm DNA fragmentation index (DFI) was detected by the sperm chromatin structure assay. Semen samples with low DFI (< 30%, control group) and high DFI (≥ 30%, experimental group) were optimized by density gradient centrifugation (DGC), and the differentially expressed proteins of obtained sperm were identified by the Sequential Window Acquisition of All Theoretical Mass Spectra Mass Spectrometry (SWATH-MS) and performed GO and KEGG analysis.

**Results:**

A total of 2186 proteins were identified and 1591 proteins were quantified, of which 252 proteins were identified as differentially expressed proteins, including 124 upregulated and 128 downregulated. These differentially expressed proteins were involved in metabolic pathways, replication/recombination/repair, acrosomal vesicles, kinase regulators, fertilization, tyrosine metabolism, etc. Western blotting results showed that the expression levels of RAD23B and DFFA proteins and the levels of posttranslational ubiquitination and acetylation modifications in the experimental group were significantly higher than those in the control group, which was consistent with the results of proteomics analysis.

**Conclusions:**

Proteomic markers of sperm with high DNA fragmentation can be identified by the SWATH-MS and bioinformatic analysis, and new protein markers and posttranslational modifications related to sperm DNA damage are expected to be intensively explored. Our findings may improve our understanding of the basic molecular mechanism of sperm DNA damage.

## Highlights

- Differentially expressed proteins in sperm could be identified by the SWATH-MS.
- The validation results of Western blotting were identical to the results of proteomics analysis.
- Proteomic markers of sperm with high DNA fragmentation could be identified by the bioinformatic analysis.
- New protein markers and posttranslational modifications related to sperm DNA damage are expected to be intensively explored.

## Background

Infertility is defined as the inability to conceive after 1 year of unprotected intercourse. Approximately 15% of couples are affected by infertility, and a male factor is responsible in about 50% of infertile couples [1]. The World Health Organization (WHO) has made valuable contributions toward interpreting and standardizing the results of semen analysis in the most recent edition of the WHO guidelines [2]. A basic semen analysis can generally evaluate the fertility status of a man [3]. However, when the parameters of basic semen analysis are normal and the man presents infertility, further sperm function tests such as sperm DNA damage, sperm acrosin activity, mitochondrial membrane potential, etc. are necessary. Although many studies suggest that sperm DNA damage may be an important reason for male infertility [4, 5], and many clinical practice guidelines note that the detection of sperm DNA fragmentation (SDF) can help clinicians assess male fertility [6–8], the exact mechanism leading to sperm DNA damage is still poorly understood.

Spermatozoa have the task of delivering an intact paternal genome to the oocyte and supporting successful embryo development. The quality of sperm DNA may affect the quality of embryos. The study of sperm function has been one of the hotspots of male infertility research. Spermatogonia undergo mitosis, meiosis and metamorphosis to form highly differentiated sperm composed of head, midsection and tail. During sperm maturation, histones in sperm nuclei are converted to protamine, and chromatin is highly concentrated. If the protein associated with sperm DNA is dysregulated, or some germ cells fail to undergo apoptosis and escape from the programmed phagocytosis process, defective mature sperm are formed, which is often manifested as increased SDF [9]. Even though semen parameters are in the normal range, the final pregnancy outcomes may be poor [10, 11]. High levels of SDF in subfertile men may affect the normal reproductive process and the health of offsprings [12]. SDF refers to the breaking of single or double strands of DNA in sperm nucleus, which tends to persist and may have a negative impact on male reproductive potential, and then on the outcomes of natural and assisted reproductive pregnancies, especially on the development of embryos implanted in assisted reproductive technology (ART) procedures [13–15]. Oleszczuk et al. [16] reported that SDF affected the rates of high-quality embryos, live birth, and miscarriage significantly. Zini et al. [17] and Kennedy et al. [18] also reported that sperm DNA damage could lead to a significant increase in the rate of miscarriage. Sperm DNA is affected by many factors, and researches on the mechanism of sperm DNA damage are still ongoing. The mature male gamete lacks the ability to repair DNA damage. Compelling evidence showed that proteins were critical in cell remodelling events, and that their abnormal expressions were associated with pronounced defects in sperm function. Proteomics has been widely used in the pathobiological study of infertility [19], and has greatly promoted our understanding of spermatogenesis. High-resolution mass spectrometry technology can decipher complex sperm protein expression features, provide insight into molecular processes associated with male infertility, and can be used to identify potential diagnostic and therapeutic biomarkers for male infertility [20, 21].

SWATH (Sequential Windowed Acquisition of all Theoretical fragments ions) is a mass spectrometry acquisition mode technology introduced in 2012, which is an extension of MS/MS-ALL technology. Through super high-speed scanning and secondary fragmentation of all peptide parent ions in the scanning region, the XIC (Extracted ion chromatography) of secondary fragmentations is directly constructed, so as to obtain complete peptide information [22]. With the help of the advanced Triple TOF 5600 plus mass spectrometry system, SWATH is a truly panoramic and high-throughput mass spectrometry technology with high quantitative accuracy and dynamic range. Based on the above, this study used the SWATH-MS technology to perform proteomic analysis on the sperm with high and low DNA fragmentation index (DFI), so as to find differential proteins, lay a foundation for exploring the mechanism of sperm DNA damage, and provide possible targets for future sperm selection.

## Methods

### Collection of semen samples

A total of 24 semen samples were collected from clinical patients by masturbation after 2–7 days of abstinence. All patients did not have obvious bad habits such as smoking, excessive drinking, staying up late, sauna, etc., use some medications, and suffer from basic diseases. After routine semen analysis was performed by a computer-aided sperm analysis (CASA) system (Beijing Suijia Medical Instrument Co., Ltd., Beijing, China), the remaining semen was used for the analysis of sperm DFI and preparation of sperm samples. The profiles of these semen samples are shown in Additional file 1: Table S1. These samples were divided into the experimental group and control group according to sperm DFI values. The patients’ ages in the experimental group and control group were 30.42 ± 3.68 and 29.17 ± 3.97 years old, respectively, and there was no significant difference between them (*P* = 0.432). The values of DFI in the experimental group (*n* = 12) were more than 30% (DFI ≥ 30%), while those in the control group (*n* = 12) were below 30% (DFI < 30%). Then, sperm were isolated from each semen sample by the density gradient centrifugation (DGC) method. Every 4 sperm samples in the experimental group and control group formed one replicate for sperm protein analysis, respectively. Three replicate samples in the experimental group were labeled as EXP1, EXP2 and EXP3, respectively, and 3 replicate samples in the control group were labeled as CON1, CON2 and CON3, respectively. This study has been approved by the Northern Jiangsu People’s Hospital ethics committee (Approval number: 2021ky068), and all patients provided informed written consent.

### Detection of sperm DFI

Sperm DFI was detected by the sperm chromatin structure assay (SCSA) [11, 23], and the corresponding kit was purchased from Zhejiang Cellpro Biotech Co., Ltd. (Ningbo, China). First, appropriate volume of semen were added into 0.1 ml of solution A (TNE buffer, sperm dilution) and mixed. Then, 0.2 ml of solution B (acid solution of 0.1% Triton X-100, 0.15 mol/l NaCl, and 0.08 mol/l HCl, pH 1.2) were added and mixed. After standing for 30 s, 0.6 ml of acridine orange (AO) staining solution (6 μg/ml AO, 37 mmol/l citric acid, 126 mmol/l Na_2_HPO_4_, 1 mmol/l Na_2_EDTA, 0.15 mol/l NaCl, pH 6.0) was added and mixed. After sperm were stained for 3 min, sperm DFI was detected by a flow cytometer (FACS Calibur, BD Bioscience, San Jose, CA, USA). A minimum of 5,000 sperm were acquired, and the data were analyzed by the software (DFIView 2010 Alpha11.15, CellPro Biotech, Ningbo, China). Sperm DFI was expressed as the percentage of sperm with fragmented DNA compared to the total number of sperm. The variability of the replicate DFI measures was less than 5%.

Since sperm used for protein analysis were selected by DGC to remove non-sperm cells, was there still a difference in sperm DFI after DGC between the experimental group and control group? To verify this, we compared sperm DFI of 6 sperm samples in each group before and after DGC.

### Preparation of sperm samples

Sperm samples were prepared by the DGC method according to the report of de Mateo et al. [24]. In brief, SpermGrad lower layer (90%), upper layer (45%) and SpermRinse solutions (Vitrolife, Sweden) were taken out from a refrigerator and recovered to room temperature for further use. First, 1 ml of SpermGrad lower layer (90%) solution was added into a 15-ml centrifuge tube, and then 1 ml of SpermGrad upper layer (45%) solution was gently added on the surface of SpermGrad lower layer (90%) solution. Next, normally liquefied semen was slowly added, and a clear interface between semen and gradient solutions could be seen. After 20 min of centrifugation at 400*g*, the upper liquids were carefully aspirated away using a pipette, and sperm sediments were transferred into a new centrifuge tube with the help of 3 ml of SpermRinse solution. The mixture was blown up and down slowly, and then centrifuged for 10 min at 200*g*. Next, the upper liquids were carefully aspirated away using a pipette. The obtained sperm were stored at − 80 °C and used for the extraction of proteins.

### Preparation of sperm proteins and peptides

The process of sperm protein library building mainly includes protein extraction, protein quantification, desalting, mass spectrometry, database retrieval, etc.

First, sperm samples were incubated in lysis buffer (7 mol/l urea, 2 mol/l thiourea, 4% sodium dodecyl sulfate, 40 mmol/l Tris–HCl, pH 8.5) containing 1 mmol/l phenylmethylsulfonyl fluoride (PMSF) and 2 mmol/l ethylene diamine tetraacetic acid (EDTA) for 5 min, and then 10 mmol/l dithiothreitol (DTT, final concentration) was added to the sample. The suspension was sonicated for 10 min on ice and then centrifuged at 16,000*g* for 20 min at 4 °C. The obtained supernatant was mixed with 4 volumes of precooled acetone and incubated for 2 h at − 20 °C. Then, the solution was centrifuged at 16,000*g* for 20 min at 4 °C, and the obtained protein pellets were air-dried and resuspended in 8 mol/l urea/100 mmol/l tetraethylammonium bromide (TEAB) solution (pH 8.0). The sperm protein samples were reduced for 30 min with 10 mmol/l DTT at 56 °C, and alkylated for 30 min in the dark with 50 mmol/l iodoacetamide (IAM) at room temperature. Next, four volumes of precooled acetone were added and incubated for 2 h at − 20 °C. Then, the solution was centrifuged at 16,000*g* for 20 min at 4 °C, and the obtained protein pellets were air-dried and resuspended in 8 mol/l urea/100 mmol/l TEAB solution (pH 8.0). The total protein concentration of the obtained solution was measured using the Bradford method. The protein precipitates were collected and dried, and then stored at − 80 °C until for further analysis.

The obtained sperm protein solution was further diluted with 5 volumes of 100 mmol/l TEAB (pH 8.0). Then, trypsin was added at an enzyme-protein ratio of 1:50 (w/w), and sperm proteins were digested overnight at 37 °C. The peptide sample was dissolved in 2% acetonitrile/0.1% formic acid solution and analyzed with Triple TOF 5600 plus mass spectrometer coupled with Eksigent nanoLC system (AB SCIEX, USA). First, peptide solution was added to the C18 capture column (3 μm, 300 μm × 0.5 mm, AB Science, USA). Then, gradient elution was performed on the C18 analytical column (3 μm, 75 µm × 150 mm, Welch Materials, Inc., USA) with a time gradient of 60 min and a flow rate of 300 nl/min. Their mobile phases were buffer A (2% acetonitrile/0.1% formic acid/98% H_2_O) and buffer B (98% acetonitrile/0.1% formic acid/2% H_2_O), respectively. For information-dependent collection (IDA), the first-order mass spectrum (MS1) was scanned with an ion accumulation time of 250 ms, and the second-order mass spectrum (MS2) of 30 precursor ions was collected using an ion accumulation time of 50 ms. The MS1 spectrum was collected in the range of 350–1200 m/z, and the MS2 spectrum was collected in the range of 100–1500 m/z. The dynamic elimination time of precursor ions was set as 15 s. The mass spectrometry data were analysed using ProteinPilot 4.5 software (July 2012; AB Sciex). Spectral library generation and SWATH data processing were performed with the Peakview version 2.2 software.

### Western blotting

The reliability of proteomics could be validated by Western blotting. Two differentially expressed proteins (DFFA and RAD23B) and two major protein modifications (ubiquitination and acetylation) were selected for Western blotting.

Briefly, 20 µg of protein was separated by 12% polyacrylamide gel electrophoresis (concentrated gel at 80 V for 50 min and separated gel at 120 V for 2 h). Subsequently, the gels were stained with Coomassie blue dye or proteins were transferred to polyvinyl difluoride (PVDF) membranes (Millipore, Bedford, Mass, USA) at 30 V overnight at 4 °C using the Mini TransBlot transfer unit (Bio-Rad, Hercules, CA, USA). Then, the membranes were blocked in tris-buffered saline (TBS) containing 0.1% Tween-20 and 5% nonfat dry milk for 60 min at room temperature, and incubated with antibodies against acetyllysine (PTM-101, 1:1000, PTM BIO, Hangzhou, China), ubiquitins (PTM-1106, 1:1000, PTM BIO, Hangzhou, China), DFFA (ab108924, Abcam, USA) and RAD23B (12,121–1-AP, Proteintech, China) overnight at 4 °C, respectively. Subsequently, the membranes were washed with PBST (PBS, 0.05% Tween-20), and incubated with horseradish peroxidase-conjugated secondary antibody (1:10,000; Pierce, Rockford, IL, USA) for 1 h at room temperature. Last, the membranes were detected by the enhanced chemiluminescence.

### Functional enrichment analysis

Proteomics analysis of sperm proteins was carried out by the SWATH-MS according to previous reports [25], and then differentially expressed proteins were performed Gene Ontology (GO) analysis by the link http://geneontology.org/. All of differentially expressed proteins were assigned to their GO annotations, including biological process (BP), cellular component (CC), and molecular function (MF). Furthermore, the Kyoto Encyclopedia of Genes and Genomes (KEGG) annotations of differentially expressed proteins were obtained by the link https://www.kegg.jp. The STRING database (https://cn.string-db.org/) was used to identify the functional enrichments, and the Cytoscape software 3.5.1 was used to visualize the interaction among proteins.

### Statistical analysis

The quantitative values of proteins were mainly calculated by the peak area of the mass spectrum data. Then, the mean value of each protein in each sample group was calculated, and the median of the ratio of the sample value to the mean value was taken as the normalization factor of the sample. The differential expressions of sperm proteins between the experimental and control groups were analyzed by the DEqMS/Bioconductor package, and the candidates with a Q-value ≤ 0.05 and a |Fold change|≥ 2 were considered as differentially expressed proteins. The differences in sperm DFI and the expression levels of differential proteins between the experimental and control groups were analyzed using Student’s *t*-test of SPSS 22.0 statistical software (SPSS Inc., Chicago, IL, USA), and *P* < 0.05 was considered statistically significant.

## Results

### Comparison of sperm DFI between the experimental group and control group

There was a statistically significant difference in sperm DFI between the experimental group (DFI ≥ 30%) and control group (DFI < 30%) (40.65% *vs* 11.42%, *P* < 0.001). Regardless of before DGC (35.96% *vs* 14.70%, *P* < 0.001) or after DGC (8.83% *vs* 2.34%, *P* = 0.005), sperm DFI in the experimental group was always significantly higher than that in the control group.

### Basic analysis of SWATH-MS data

**Fig. 1:**
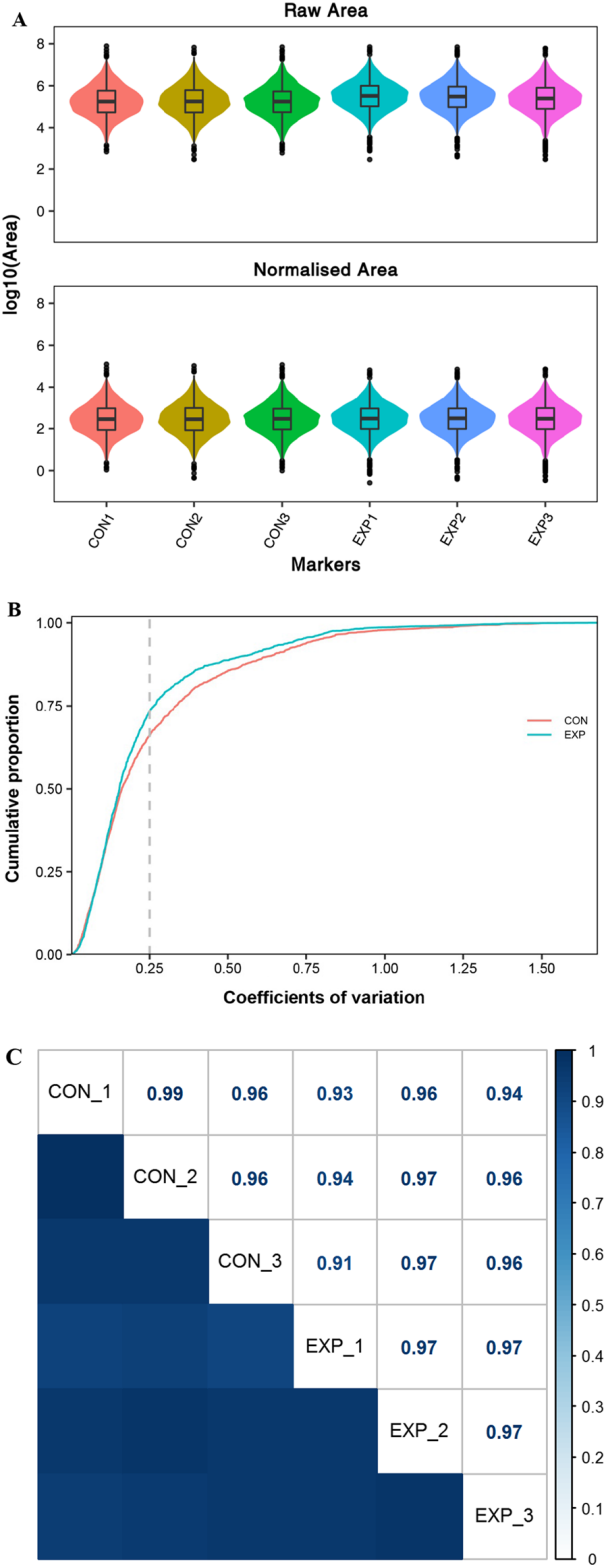
Qualitative and quantitative analysis and correlation analysis of proteins identified by the SWATH-MS technology. **A** Box plot for the distribution of protein abundance values from different samples, showing that there was good reproducibility between different samples. CON: The control group with sperm DNA fragmentation index (DFI) < 30%; EXP: The experimental group with DFI ≥ 30%. **B** Box plot for the distribution of coefficients of variation from different samples, showing that there was good reproducibility between different samples. CON: The control group with sperm DNA fragmentation index (DFI) < 30%; EXP: The experimental group with DFI ≥ 30%. **C** Box plot for Pearson correlation analysis of protein quantitative values between different samples, showing that there was good reproducibility between different samples. CON: The control group with sperm DNA fragmentation index (DFI) < 30%; EXP: The experimental group with DFI ≥ 30%. The number in the box represented the correlation coefficient between different samples. The darker the color, the greater the correlation coefficient

A total of 142,329 credible peptide spectrum matches (PSMs) were obtained by the data dependent acquisition (DDA) library based on the confidence ≥ 0.95. In our study, a total of 33,248 peptide spectra were identified, and there were 10,761 matching. Last, a total of 24,526 peptide spectra, 4,088 peptides and 1,591 proteins were quantified by the SWATH-MS technology. Moreover, good reproducibility was observed in the box plots of the distribution of protein abundance values (Fig. 1A), coefficients of variation (CV) (Fig. 1B) and Pearson coefficients (Fig. 1C).

### Analysis of differentially expressed proteins

**Fig. 2:**
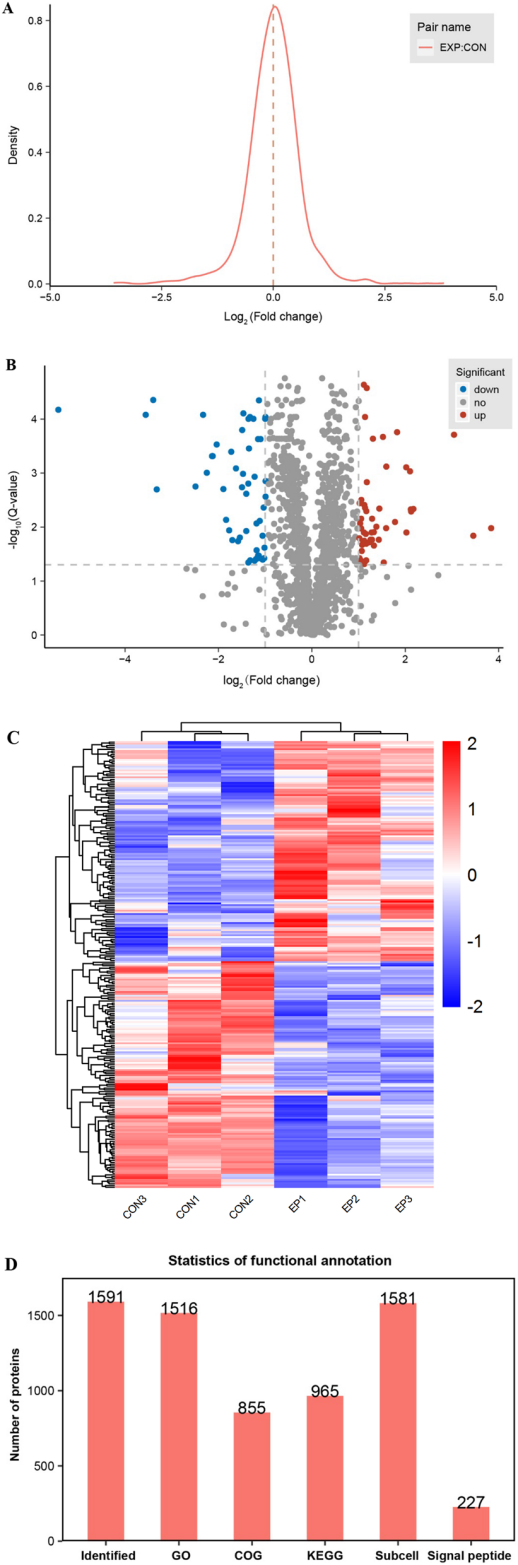
Comparison and analysis of differentially expressed proteins between the experimental group and control group. **A** Distribution of the ratios of protein abundance between the experimental group and control group. EXP: The experimental group with sperm DNA fragmentation index (DFI) ≥ 30%; CON: The control group with sperm DFI < 30%. If a log-transformed value of the fold change with base 2 is greater than 0, it indicates that the levels of differentially expressed proteins are upregulated. Conversely, the levels of differentially expressed proteins are downregulated. **B** Volcano plot of differentially expressed proteins between the experimental group and control group. The red and blue dots on both sides of the volcano plot represent significantly upregulated and downregulated proteins, respectively. **C** Hierarchical clustering heatmap of differentially expressed proteins between the experimental group and control group. EXP: The experimental group with sperm DNA fragmentation index (DFI) ≥ 30%; CON: The control group with sperm DFI < 30%. Rows represent protein clustering, and columns represent sample clustering. **D** Statistical chart of the annotation results for different functions of all quantitative proteins. GO: Gene Ontology; COG: Clusters of Orthologous Groups; KEGG: Kyoto Encyclopedia of Genes and Genomes

A total of 252 proteins with significant changes were identified, of which 124 were upregulated and 128 were downregulated. The main upregulated proteins included DFFA, USO1, IQGA1, DHX9, SC22B, PP6R1, HUWE1, RAD23B, EMAL2, ESPB1, etc. The main downregulated proteins included MPPB, ZPBP1, ATIF1, AKAP4, L37A1, ACRBP, SPESP, ATIF1, etc. The logarithm of the difference multiples was taken as base 2 to make a distribution map, which approximately obeys the normal distribution (Fig. 2A). Then, the volcano plot was drawn with Log_2_(Fold change) as the abscissa and − log_10_(Q-value) as the ordinate, and the differentially expressed proteins with |Fold change|≥ 2 and Q-value ≤ 0.05 were screened out (Fig. 2B). The results of hierarchical clustering analysis of differentially expressed proteins between the experimental group and control group showed that the samples had good repeatability (Fig. 2C). Through database alignment and software analysis, the identified quantitative proteins were subjected to GO functional annotation, Clusters of Orthologous Groups (COG) annotation, KEGG metabolic pathway annotation, subcellular localization prediction, and signal peptide prediction (Fig. 2D).

### Functional analysis of differentially expressed proteins

**Fig. 3:**
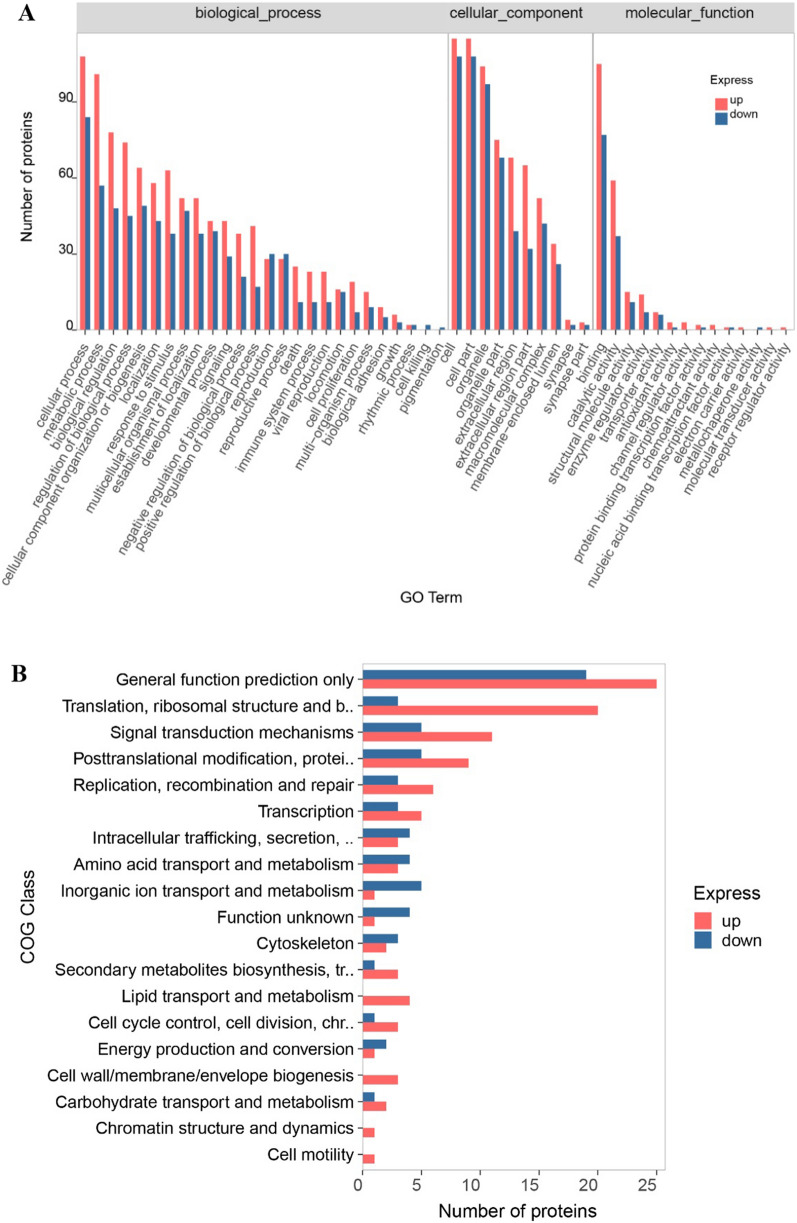

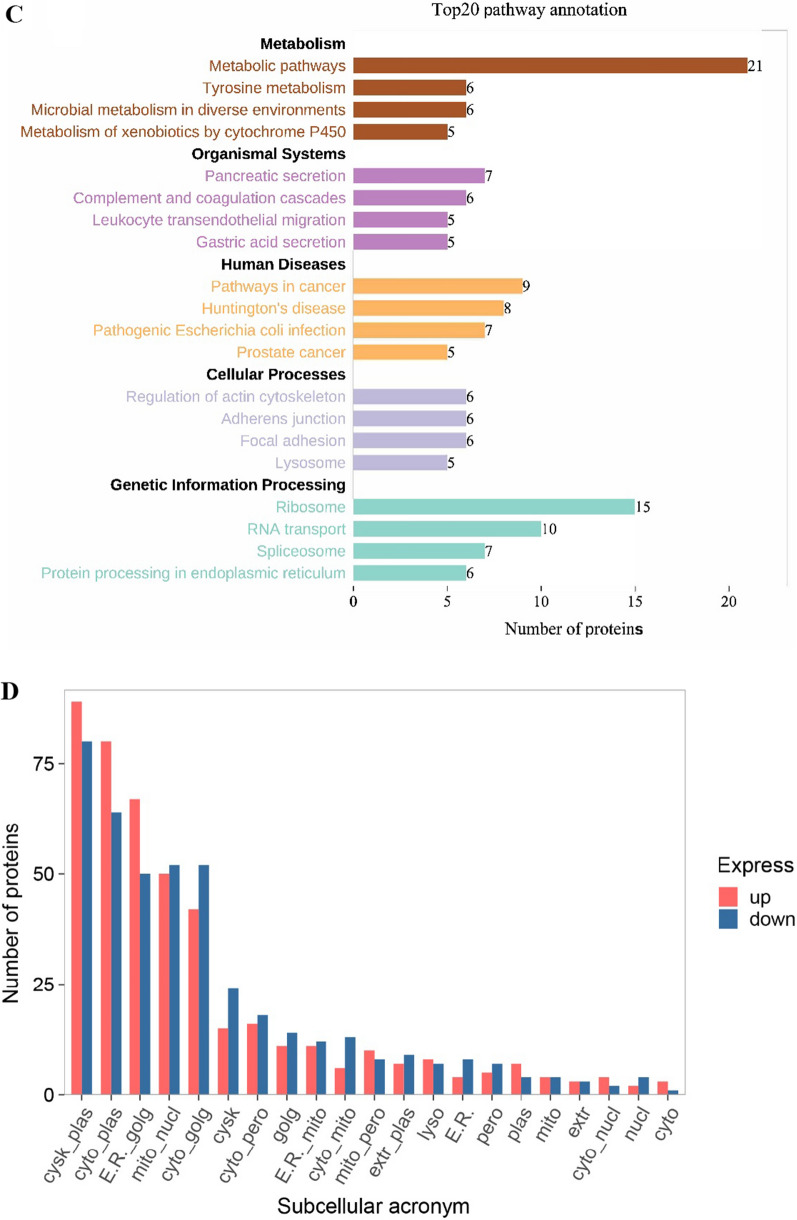
Functional annotation of differentially expressed proteins. **A** GO classification annotation histogram of differentially expressed proteins. GO: Gene Ontology. **B** Differential proteins COG classification annotation histogram of differentially expressed proteins. COG: Clusters of Orthologous Groups. **C** Statistical chart of the top 20 pathway annotation results of differentially expressed proteins. **D** Statistics of subcellular localization results

GO annotation of differentially expressed proteins mainly focused on binding, catalytic activity, structural molecule activity, antioxidant activity, cellular process, metabolic process, response to stimulus, developmental process, etc. (Fig. 3A). COG annotation was mainly in general function prediction only, translation/ribosomal structure and biogenesis, signal transduction mechanisms, posttranslational modification/protein turnover/chaperones, replication/recombination and repair, etc. (Fig. 3B). KEGG pathway analysis mainly focused on metabolic pathways, microbial metabolism in diverse environments, ribosome, RNA transport, protein processing in endoplasmic reticulum, MAPK signaling pathway, mTOR signaling pathway, phosphatidylinositol signaling system, calcium signaling pathway, regulation of actin cytoskeleton, lysosome, endocytosis, phagosome, p53 signaling pathway, etc. (Fig. 3C). Subcellular localization analysis mainly focused on the cytoskeleton-plasma, cytoplasm-plasma membrane, endoplasmic reticulum-Golgi apparatus, mitochondria-nucleus, etc. (Fig. 3D).

### GO enrichment and KEGG enrichment analysis of differentially expressed proteins

**Fig. 4:**
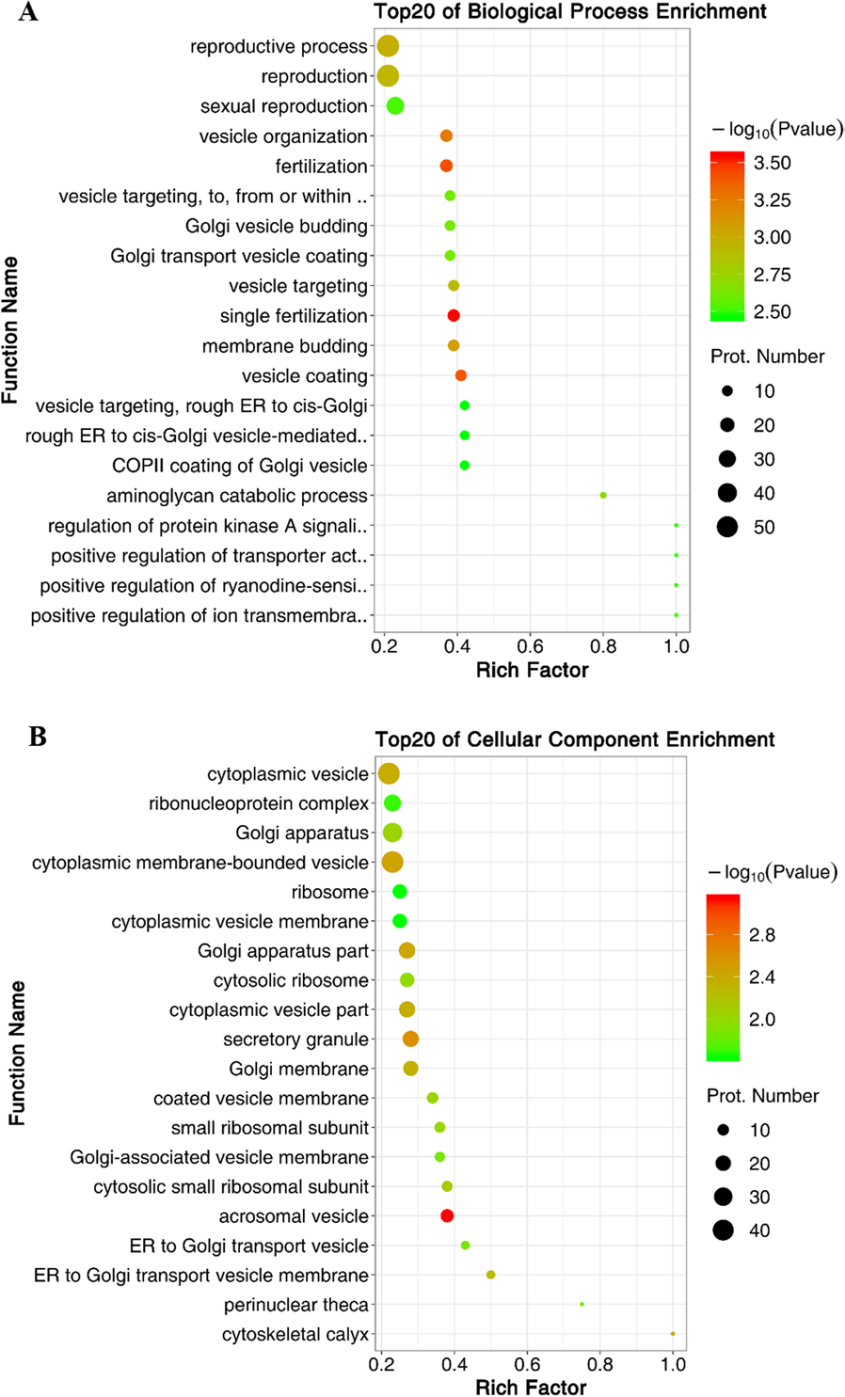

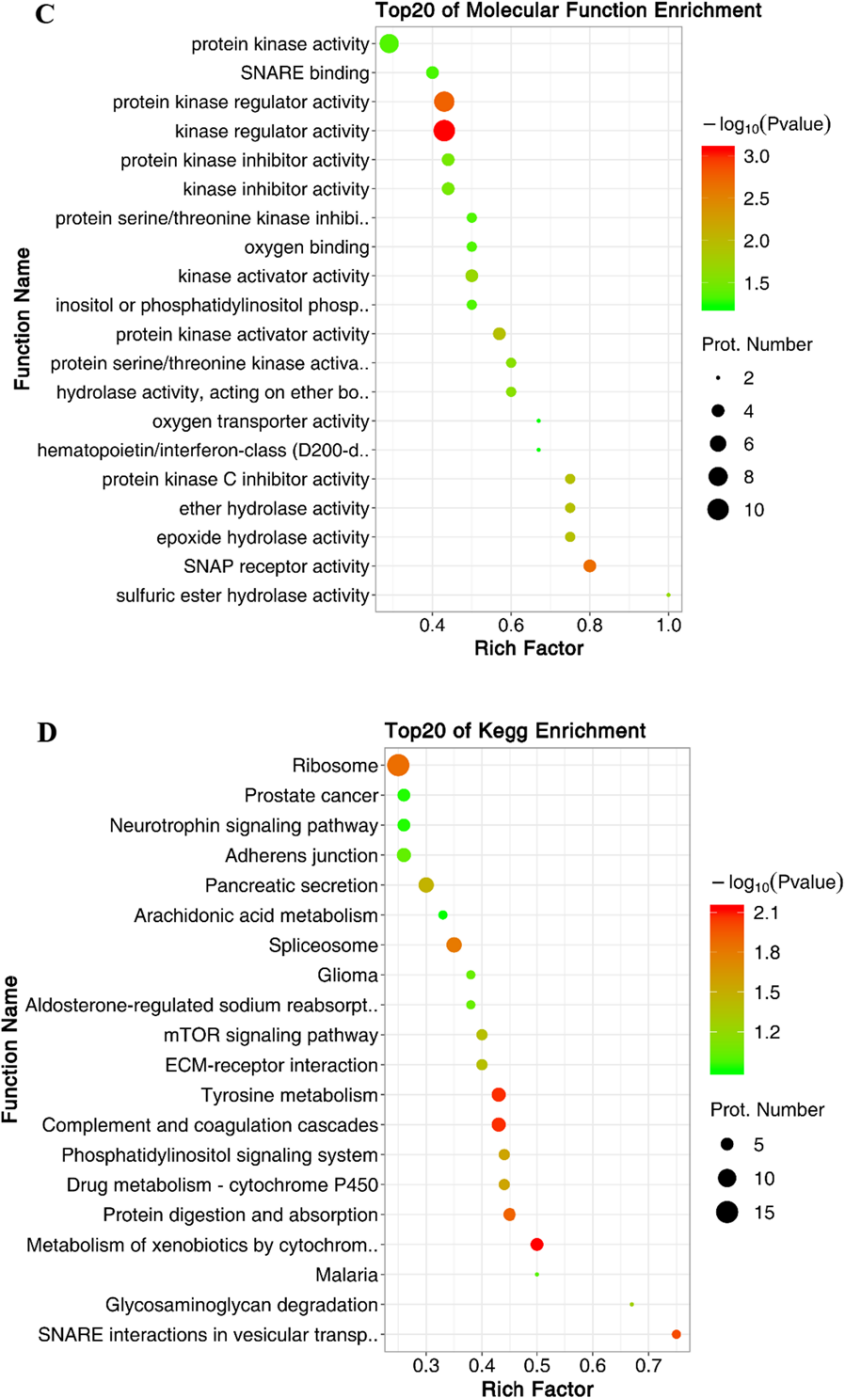
GO and KEGG pathway enrichment analysis of differentially expressed proteins. **A** Bubble charts of the top 20 biological process enrichment of differentially expressed proteins. Prot.: Protein. **B** Bubble charts of the top 20 cellular component enrichment of differentially expressed proteins. Prot.: Protein. **C** Bubble charts of the top 20 molecular function enrichment of differentially expressed proteins. Prot.: Protein. **D** Bubble chart of the top 20 KEGG pathways of differentially expressed proteins. KEGG: Kyoto Encyclopedia of Genes and Genomes; Prot.: Protein

GO enrichment analysis mainly included reproductive process, reproduction, vesicle organization, fertilization, cytoplasmic vesicle, acrosomal vesicle, kinase activity, protein kinase regulator activity, etc. (Fig. 4A–C). KEGG pathway enrichment was mainly in ribosome, mTOR signaling pathway, metabolism of xenobiotics by cytochrome, protein digestion and absorption, p53 signaling pathway, lysosome, apoptosis, peroxisome, phagosome, phosphatidylinositol signaling system, nucleotide excision repair (nucleoside acid excision repair), etc. (Fig. 4D).

### Differentially expressed proteins were confirmed by Western blot and the changes of sperm protein modifications

**Fig. 5:**
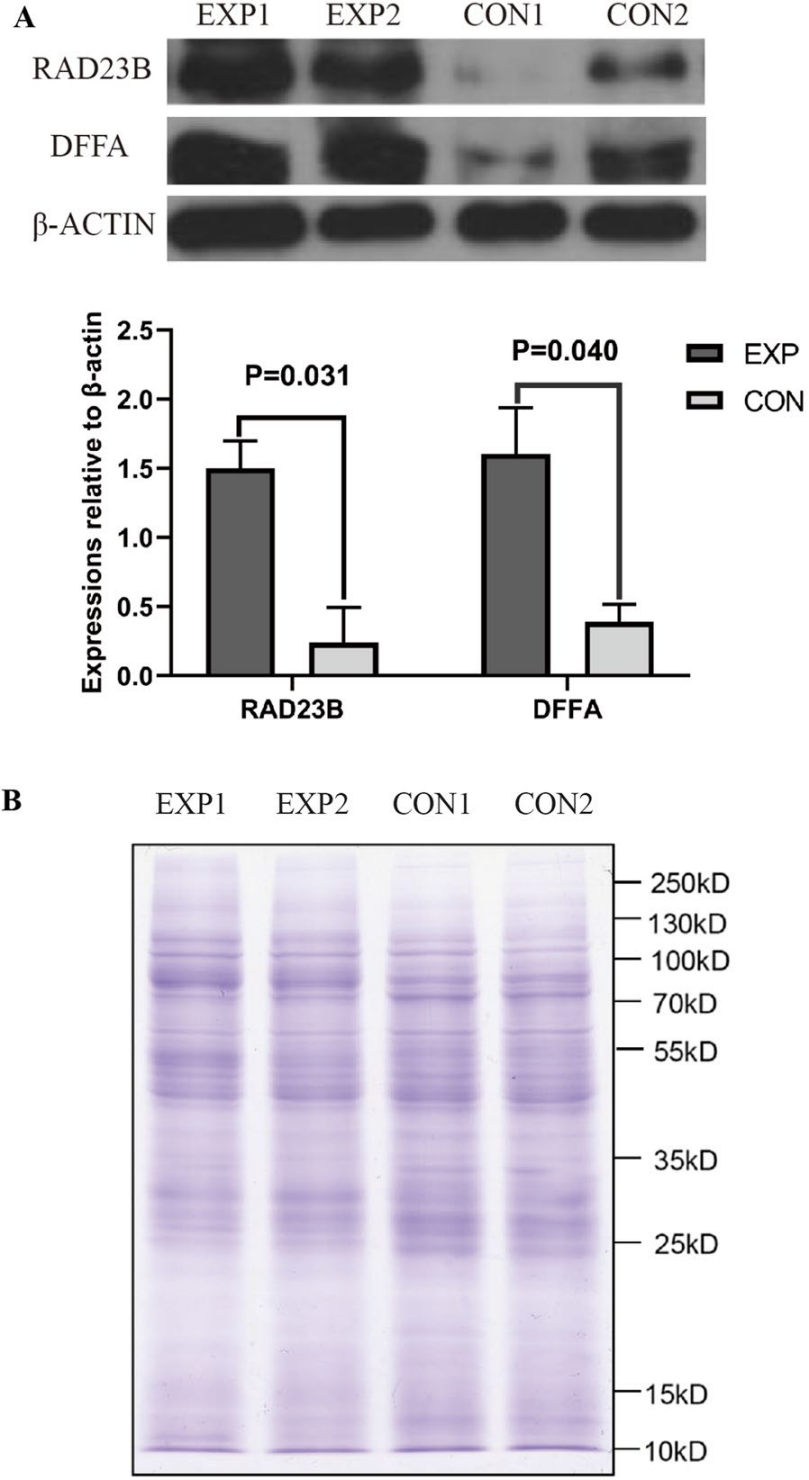

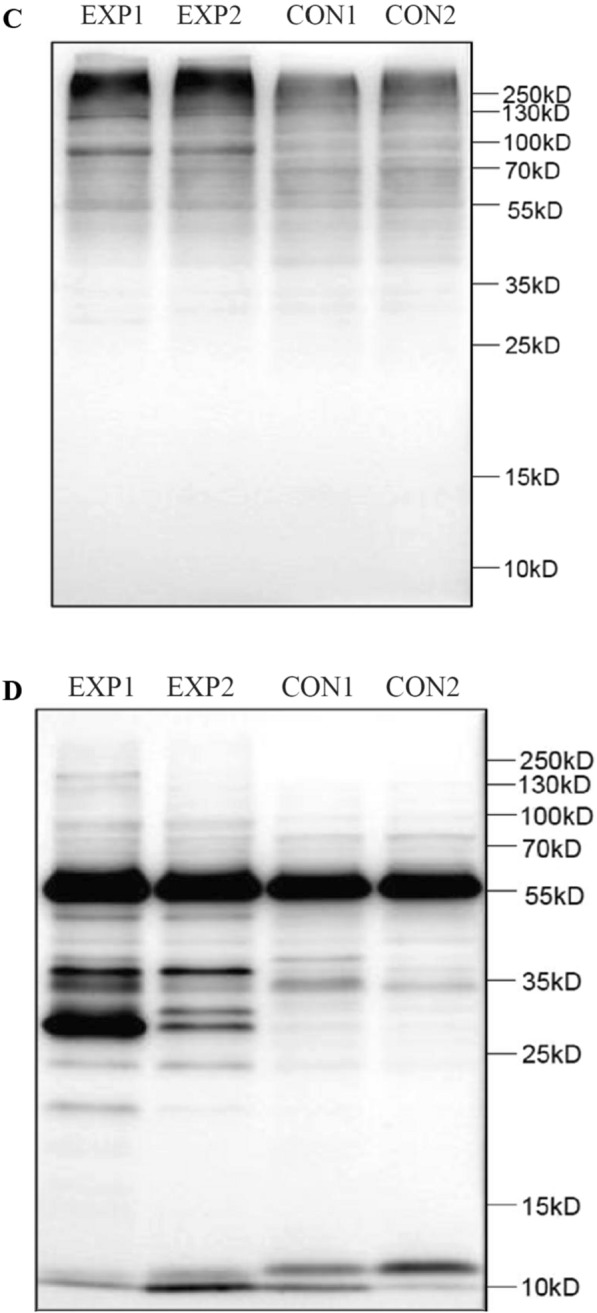
Comparisons of differentially expressed proteins and two protein modifications between EXP and CON groups. EXP: The experimental group with sperm DNA fragmentation index (DFI) ≥ 30%; CON: The control group with sperm DFI < 30%; *n* = 2 for each group. **A** Western blot showed that the expression levels of RAD23B and DFFA in the experimental group were significantly higher than those in the control group, which were consistent with the results of proteomic analysis. **B** Sperm proteins were separated by 12% of polyacrylamide gel electrophoresis and stained by Coomassie brilliant blue, and there was no obvious changes in sperm protein levels between the experimental group and control group. **C** Sperm proteins were separated by 12% of polyacrylamide gel electrophoresis and detected with antibodies against ubiquitins, and the results showed that the ubiquitination modification of sperm proteins at approximately 100 kD in the experimental group was significantly higher than that in the control group. **D** Sperm proteins were separated by 12% of polyacrylamide gel electrophoresis and detected with antibodies against acetyllysine, and the results showed that the acetylation modification of sperm proteins at 20–45 kD in the experimental group was significantly higher than that in the control group

Western blot showed that the expression levels of RAD23B and DFFA in the experimental group were significantly higher than those in the control group, which were consistent with the results of proteomic analysis (Fig. 5A). Meanwhile, two kinds of major protein modifications of spermatozoa were detected. It was found that there was no obvious changes in sperm protein levels between the experimental group and control group (Fig. 5B). However, the ubiquitination modification of sperm proteins at approximately 100 kD and acetylation modification at 20–45 kD in the experimental group were significantly higher than those in the control group (Fig. 5C, D).

## Discussion

Currently, the selection of ART such as intrauterine artificial insemination (IUI), in vitro fertilization-embryo transfer (IVF-ET) and intracytoplasmic sperm injection (ICSI) is mainly based on the results of routine semen analysis, including sperm concentration and motility in the raw semen samples as well as after DGC and/or swim-up. However, in clinical practice, even if ICSI or IVF is performed using high-quality sperm after semen optimization, the final clinical outcomes still show significant differences [26–29], which may be related to sperm DNA damage. Therefore, studying the molecular biological mechanism of sperm DNA fragmentation during spermatogenesis can provide new ideas for searching for proteins which may affect embryonic development and are related to sperm DFI.

Although sperm DNA fragmentation levels in sperm samples with high DFI were significantly reduced after the optimization of DGC, it was found that sperm DNA fragmentation levels in the experimental group (DFI ≥ 30%) after DGC were still significantly higher than that in the control group (DFI < 30%), which was consistent with the results of Wang et al. [30]. Subsequently, the SWATH-MS technology was used to compare sperm proteins between the two groups, and 252 differentially expressed proteins were obtained, of which 124 were significantly upregulated and 128 were significantly downregulated. GO analysis results of differentially expressed proteins showed that they were mainly associated with translation, ribosome structure and biogenesis, signal transduction mechanism, replication/recombination and repair, transcription, amino acid transport and metabolism, secretion and vesicle transport, etc. KEGG pathway analysis showed that differentially expressed proteins were mainly related to the tyrosine metabolism pathway, MAPK signaling pathway, mTOR signaling pathway, phosphatidylinositol signaling system, etc. The molecular functions annotated by GO enrichment analysis were mainly concentrated in binding proteins, catalytic activity, structural molecular activity, enzyme regulator activity, antioxidant activity, etc. The proteins annotated in biological functions were mainly concentrated in cellular processes, metabolic processes, biological regulation, stress response, etc. Studies have shown that the overproduction of reactive oxygen species (ROS) can lead to oxidative stress-induced DNA damage, showing an increase in sperm DNA fragmentation levels [31]. Oxidative stress is a condition caused by an imbalance between the concentrations of oxidants and antioxidants [32], and the proteins related to antioxidant activity obtained in this study may play a role in the pathway of sperm oxidative stress leading to DNA damage. The environmental information pathways mainly include the MAPK signaling pathway, mTOR signaling pathway, phosphatidylinositol signaling system, calcium signaling pathway, etc. Studies have shown that sperm DFI is related to lipoprotein particle remodelling and regulation, fatty acid binding and other functions [33]. The differentially expressed proteins related to the nucleotide excision pathway and fatty acid metabolism pathway screened in this study may play an important role. The KEGG pathway enrichment analysis results of differentially expressed proteins showed that they mainly focused on the p53 signaling pathway, lysosome, cell cycle, apoptosis, peroxisome, phagosome, phosphatidylinositol signaling system, mTOR signaling pathway, etc.

Two differentially expressed proteins were selected for Western blotting, and the results showed that the expression levels of RAD23B (RAD23 homologue B) and DFFA (DNA fragmentation factor subunit alpha) in the experimental group were significantly higher than those in the control group, which was consistent with the results of proteomics analysis. RAD23B is a homolog of yeast ultraviolet excision repair protein RAD23 [34], wherein RAD23B and XPC (xeroderma pigmentosum complementation group C) form an XPC-RAD23B complex, which plays a key role in the recognition of DNA damage in genomic nucleotide excision repair (NER) by identifying and interacting with unpaired bases in the DNA strand [35, 36]. Moreover, RAD23B has been confirmed to be expressed in human testis [37]. RAD23B has ubiquitin-like domains at its N-terminal and two ubiquitin-related domains at its central and C-terminal regions, and its binding protein partners are involved not only in DNA repair but also in ubiquitin-dependent protein degradation, transcriptional regulation and cell cycle control [38, 39]. RAD23B may play an important role in sperm DNA damage and repair. DFFA is a factor directly related to DNA fragmentation [40–42]. When sperm DNA breaks are being repaired, the expression level of RAD23B increases, which leads to the accumulation of DFFA.

Posttranslational modifications (PTMs) are key regulators of biological system responses to external stimuli, which regulate protein conformational changes, activity and function, and are involved in nearly all of cellular pathways and processes [43]. Each modification originates from a specific local physiological or pathobiological process [44]. The identification of protein posttranslational modifications is the basis for understanding cellular and molecular mechanisms. There is evidence that acylation [45, 46] and ubiquitination [47] in proteins play key roles in spermatogenesis, sperm maturation and fertilization process. Therefore, we evaluated the two major protein modifications in the optimized sperm. It was found that there was no significant difference in the expression levels of proteins between the experimental group and control group. However, the ubiquitin modification levels of sperm proteins at approximately 100 kD and acetyllysine modification levels at 20–45 kD in the experimental group were significantly higher than those in the control group, suggesting that the posttranslational ubiquitination and acetylation modifications of sperm proteins may play an important role in the mechanism of sperm DNA damage.

## Conclusions

Male subfertility is a complex and multifactorial disorder, and its etiology is still unknown. Sperm DNA damage may be an important reason for male subfertility. In this study, differentially expressed proteins of sperm between the experimental group (DFI ≥ 30%) and control group (DFI < 30%) were successfully obtained by the SWATH-MS technology, and the results of proteomics analysis were further validated by the Western blotting of RAD23B and DFFA proteins and posttranslational ubiquitination and acetylation modifications. Our findings may improve our understanding of the basic molecular mechanism of sperm DNA damage, while the detailed mechanism leading to sperm DNA damage needs to be further explored.

## Data Availability

The datasets used and/or analysed during the current study are available from the corresponding author on reasonable request.

## Abbreviations

WHO: World Health Organization
SDF: Sperm DNA fragmentation
ART: Assisted reproductive technology
SWATH-MS: Sequential Window Acquisition of All Theoretical Mass Spectra Mass Spectrometry
DFI: DNA fragmentation index
DGC: Density gradient centrifugation
PTMs: Posttranslational modifications
GO: Gene Ontology
COG: Clusters of Orthologous Groups
KEGG: Kyoto Encyclopedia of Genes and Genomes
CC: Cellular component
MF: Molecular function
BP: Biological process
CON: The control group
EXP: The experimental group
ROS: Reactive oxygen species
